# Basic Laparoscopic Skills Training Is Equally Effective Using 2D Compared to 3D Visualization: A Randomized Controlled Trial

**DOI:** 10.3390/jcm9051408

**Published:** 2020-05-10

**Authors:** Eliana Montanari, Richard Schwameis, Nikolaus Veit-Rubin, Lorenz Kuessel, Heinrich Husslein

**Affiliations:** Department of Gynecology and Obstetrics, Medical University of Vienna, Waehringer Guertel 18-20, 1090 Vienna, Austria; eliana.montanari@meduniwien.ac.at (E.M.); richard.schwameis@meduniwien.ac.at (R.S.); nikolaus.veit-rubin@meduniwien.ac.at (N.V.-R.); lorenz.kuessel@meduniwien.ac.at (L.K.)

**Keywords:** basic laparoscopic skills (BLS), depth perception, laparoscopy, three-dimensional visualization, two-dimensional visualization, Fundamentals of Laparoscopic Skills (FLS), training

## Abstract

Reduced depth perception due to two-dimensional (2D) visualization of a three-dimensional (3D) space represents a main challenge in acquiring basic laparoscopic skills (BLS); 3D visualization might increase training efficiency. This study aimed to assess whether BLS training on a standard box trainer using 2D is at least equally effective compared to 3D. Medical students were randomized to training of Fundamentals of Laparoscopic Surgery (FLS) tasks using either 2D or 3D for four weeks. Baseline and post-training tests were performed using the assigned visualization modality. Data of 31 participants were analyzed (*n* = 16 2D, *n* = 15 3D). Baseline test scores did not differ significantly between groups; only at the peg transfer task and total scores, the 3D group performed better than the 2D group. All scores improved significantly in both groups, with post training scores not differing significantly between groups. Non-inferiority of 2D compared to 3D was demonstrated for total score improvement and improvement in all individual FLS tasks except for suturing with extracorporeal knot tying. Post training test performance did not change significantly when changing to the unfamiliar modality. In conclusion, BLS training using standard 2D is at least equally effective as with 3D, without significant disadvantages when changing to the other modality.

## 1. Introduction

Due to its numerous advantages compared to laparotomy, laparoscopy has become the standard operative technique for an increasing number of surgical procedures [1]. However, compared to open surgery, laparoscopy is more difficult to learn [2]. It requires specific psychomotor skills and surgeons have to work in a three-dimensional (3D) space using two-dimensional (2D) imaging, which leads to reduced depth perception [3]. In order to overcome the challenge of 2D imaging, 3D visualization was developed and improved over the years [4]. Today, most hospitals have not yet adopted 3D systems as a standard equipment, because they are more expensive and so far, studies analyzing the possible advantages of 3D compared to 2D visualization have shown conflicting results [5]. Additionally, different side effects related to 3D visualization, such as dizziness, nausea, discomfort, eye strain, or tiredness have been reported [4]. However, more recent studies using third-generation 3D equipment show improved performance and a reduction in surgical time and errors [4,5]. This seems to be true especially for more complex surgical procedures. For example, a randomized trial evaluating the time needed for laparoscopic bypass showed that total operative time and time needed for the hand-sewn gastrojejunal anastomosis were significantly shorter using a 3D imaging system compared to conventional 2D visualization [6]. Reduced operation time using 3D compared to 2D was also seen for other surgical procedures such as total laparoscopic hysterectomy, for which a retrospective study analyzing hysterectomies for large uteri over 500 g found not only a reduced total operative time, but also lower blood loss and complication rates in the 3D compared to the 2D group [7].

In the educational setting with simpler surgical tasks, no significant difference between the two visualization modalities was found; for example, a randomized controlled study evaluating the laparoscopic closure of the vaginal cuff during total laparoscopic hysterectomy by surgeons-in-training could not show any significant difference between 2D and 3D systems in the time needed for completion of this surgical step, and also the surgeons’ subjective rating of the “ease of cuff closure” did not differ between groups [8].

Apart from its application in the operating room (OR), the question about the most convenient visualization modality also arises for basic laparoscopic skills (BLS) training in a simulation environment. Training of such skills outside the OR without putting patient’s safety at risk is gaining increasing importance [9,10]. Various simulation models have been developed and studied for this purpose, including virtual reality trainers, commercially available standard box trainers and inexpensive home-made laparoscopic box trainers [10,11]. Almost all of these simulation models are based on 2D visualization. However, since most of the difficulties in acquiring BLS arise from the challenges of 2D to 3D orientation and the resulting difficulties in depth perception, it would be of great interest whether 3D might have advantages over conventional 2D imaging for BLS training, thereby shortening learning curves and increasing efficiency. Most of the studies published on the comparison of 3D and 2D visualization, including those evaluating simulation settings, had limitations such as use of first- or second-generation 3D techniques, lack of consideration of prior laparoscopic experience, evaluation of nonstandardized procedures, or analysis of subjective data [5].

The aim of this study was to investigate whether practicing on a standard laparoscopic box trainer using 2D visualization is at least equally effective compared to 3D in terms of BLS improvement, by means of a randomized controlled trial with last-generation 3D equipment, selected participants with limited laparoscopic experience, and using standardized and validated tasks. Moreover, the performance after changing visualization modality was evaluated. This information could be valuable for selection of optimal equipment in simulation centers to obtain the highest training results.

## 2. Materials and Methods

This randomized controlled trial (ClinicalTrials.gov ID: NCT03763903) was carried out at the Surgical Skills Training Center of the Department of Gynecology and Obstetrics, Medical University of Vienna, Austria [12], between November 2018 and December 2018. Participants were randomized to one of the following two groups: training on a standard laparoscopic box trainer (1) using 2D visualization or (2) using 3D visualization.

Medical students with limited experience in laparoscopic surgery were recruited from the Medical University of Vienna. Exclusion criteria were regular practice on a box trainer over the last 12 months or previous performance of any laparoscopic operation as the primary surgeon. Written informed consent was obtained from all participants. A unique study identification number was assigned to each participant and baseline demographic data were collected at the time of consent. The need for ethical approval for this study was waived by the Ethics Committee of the Medical University of Vienna (IRB number: 1555/2018).

Training sessions took place twice a week for four weeks with each training session lasting a maximum of one hour. Participants performed the following four Fundamentals of Laparoscopic Surgery (FLS) tasks: (1) peg transfer, (2) pattern cutting, (3) suturing with extracorporeal knot tying, and (4) suturing with intracorporeal knot tying [13]. At the beginning of the study, prior to the first training session, an instruction video demonstrating the four FLS tasks was shown to all participants, followed by a baseline test for each of the four tasks on a standard pelvic trainer using the assigned visualization modality. During the training sessions, surgical instructors were present to provide feedback and supervise the participants. Participants were asked to complete five repetitions per session of the peg transfer and the pattern cutting task, whereas for both suturing tasks, participants were instructed to practice until a self-perceived improvement was noted, with a maximum overall time limit of one hour for each training session. Every training session consisted of two of the four tasks, of which one was a suturing task. At the end of the study, each participant performed two post training tests, the first using the assigned visualization modality and the second using the other modality. Both post training tests were carried out in an identical way to the baseline test.

During the study period, participants performed the four FLS tasks on a standard laparoscopic box trainer using either 2D or 3D visualization, respectively. In the 2D group, Visera Elite OTV-S190 processors, CLV-S190 light sources, and ENDOEYE HD II, 10 mm, 30° cameras (WA50042A) were used; for the 3D group, Visera Elite II OTV-S300 processors with integrated LED light sources, and ENDOEYE 3D, 10 mm, 30° cameras (WA50082A) were used (Olympus Austria Ges.m.b.H, Vienna, Austria). The cameras were fixed in order to avoid possible bias arising from differences in camera navigation.

The baseline and the post training assessments were completed by each participant performing each of the four FLS tasks once. For the baseline test, participants were allowed to become familiar with the box trainer by performing half of the first task (peg transfer) once prior to the assessment. Regarding the post training assessments, each participant performed each of the four FLS tasks once using the assigned visualization modality first. Afterwards, they changed to the other visualization modality and performed the four FLS tasks again in an identical way.

Performance of the four FLS tasks was assessed according to the time needed to complete the task as well as the accuracy of task performance using the FLS scoring system, as previously described [13]. One staff surgeon, proficient in laparoscopy and with significant experience in surgical training using FLS tasks, carried out the assessment. The improvement of performance for the total score and for each FLS task was calculated by subtracting the baseline test scores from the post training test scores of the assigned visualization modality. The primary outcome measure was the improvement in total scores within the assigned modality. Additionally, score improvement for the four individual FLS tasks was chosen as a secondary outcome measure. Furthermore, the performance after changing to the other visualization modality was evaluated.

Sample size calculation was performed for a non-inferiority trial with a continuous outcome. We considered a non-inferiority margin of 10% of the mean improvement in total scores after training of the 3D group to be the minimum clinically significant difference, and calculated the required sample size applying a single-tailed alpha of 0.025 and a power of 0.90. Calculation was based on previous results for the FLS tasks of novice trainees showing a mean improvement in total scores of 342 points with a standard deviation (SD) of 24 points [14]. According to the considerations made above, 12 participants were required in each training arm. Anticipating a dropout rate of 20%, a minimum of 15 participants had to be included per study group.

For randomization, an allocation ratio of 1:1 was used. Participants were consecutively randomized according to the concealed sequence of a computer-generated randomization plan by one of the research team members into one of the two groups by means of sealed envelope technique. No masking was used since the FLS scoring system is standardized and objective.

Data were analyzed according to the intention-to-treat principle and assessed using descriptive statistics. The Fisher exact test was used for comparison of nominal data. For normally distributed data, the *t*-test for independent samples was performed for comparisons between groups. Data not following a normal distribution were compared using nonparametric tests, using the Wilcoxon-signed-rank test for comparisons within groups and the Mann–Whitney–U test for those between groups. Two-sided *p*-Values < 0.05 were considered statistically significant.

For the analysis of the primary outcome (i.e., total score improvement) and of individual task score improvement, non-inferiority was determined if the lower bound of the two-sided 95% confidence interval (according to the *t*-test for independent samples) of the mean difference in improvements did not exceed the non-inferiority margin (i.e., 10% of the mean improvement in the 3D group). Data were analyzed using IBM SPSS version 21.0 (IBM Corp., Armonk, NY, USA).

## 3. Results

### 3.1. Participants

Thirty-two medical students were recruited and randomized to either 2D (*n* = 16) or 3D (*n* = 16) visualization. One participant of the 3D group dropped out of the study after the second training session. All other participants completed the study and were included in the final analysis. Baseline demographic data are shown in Table 1. Participants in both groups were comparable regarding year of medical school, sex, handedness, assistance in laparoscopic surgeries, box trainer simulation training experience, video game experience, playing of a musical instrument, or previous virtual reality trainer experience (Table 1).

### 3.2. Baseline Test, Post-Training Test and Score Improvement

Baseline test scores for three of the individual FLS tasks (pattern cutting, suturing with extracorporeal knot tying, and suturing with intracorporeal knot tying) did not differ statistically significantly between the 2D and the 3D group, whereas participants in the 3D group scored better than those in the 2D group at the peg transfer task and had higher total scores (Table 2). Total scores as well as the scores for all four tasks separately improved significantly in both groups from the baseline test to the post training test, using the respective assigned visualization modality (Table 2). Post-training test total scores and single task scores using the respective assigned modality did not differ significantly between groups (Table 2). Non-inferiority of training using 2D compared to 3D could be shown for the improvement in total scores and all individual FLS tasks except for the suturing with extracorporeal knot tying task (Table 3).

### 3.3. Analysis after Exclusion of Suturing with Extracorporeal Knot Tying Task Scores

As predefined, for both suturing tasks, a maximum time (and therefore 0 points) was assigned for this exercise if the drain was avulsed [13]. At the post-training tests, a drain avulsion occurred in six cases, only at the suturing with extracorporeal knot tying task, whereas this never happened at the suturing with intracorporeal knot tying task. As drain avulsion occurred at different time points during the exercise between the participants (in some cases at the end of the exercise and in other cases at the beginning, even before tying the first knot), scoring all of these cases equally with 0 points seemed not appropriate. These “0-scores” increased the standard deviation greatly without reflecting the real standard deviation in task performance between participants. For these reasons, the analyses were repeated after excluding the “suturing with extracorporeal knot tying” task. In this case, not only non-inferiority, but also superiority of training using 2D compared to 3D could be demonstrated for the improvement in total scores (2D group mean total score improvement ± SD: 206.1 ± 17.1 points; 3D group: 174.8 ± 39.5 points; *p* = 0.011 (with 95% CI of 8.1–54.5) using the *t*-test for independent samples; non-inferiority margin of −17.5 points). All other results remained unaffected.

### 3.4. Performance after Changing from the Assigned to the Other Visualization Modality

For the 2D group, there were no statistically significant differences in total scores or single task scores between the post training test using the assigned visualization modality and the post training test using the other modality (Wilcoxon-signed-rank test, *p* > 0.05 for all scores). For the 3D group, only when including the suturing with extracorporeal knot tying task, higher scores were found for this task and the total score at the 2D compared to the 3D post-training test (*p* = 0.008 and *p* = 0.041, respectively), whereas there were no differences for the other three tasks. However, when the suturing with extracorporeal knot tying task was excluded from the analyses, the difference in total scores between the post training test using the 3D and the 2D modality vanished.

Additionally, possible differences in post training test performance between groups using the 2D modality were evaluated with regard to total score and single task scores. None of the scores differed significantly between groups. The same was true for the assessment of possible differences in post training test performance between groups using 3D (Mann–Whitney–U test, *p* > 0.05 for all scores).

### 3.5. Peg Transfer Task Score Development during the Training Period

**Figure 1:**
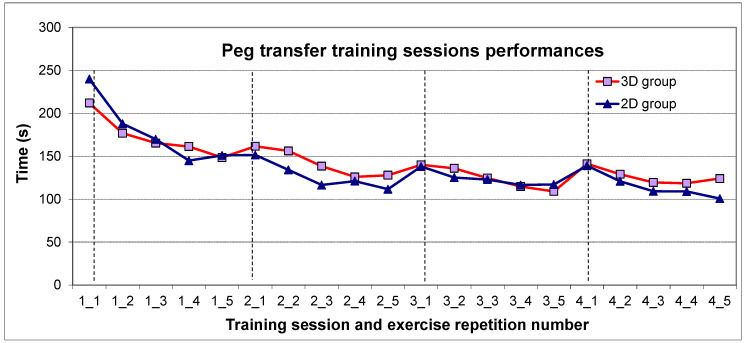
Peg transfer task score development over the four training sessions. The mean time for task completion in seconds is shown for each repetition of the peg transfer task at each training session. The red line represents the values for the participants of the 3D group and the blue one, those for the participants of the 2D group. The vertical lines mark the first repetition at each training session. On the *x*-axis, the training session and the respective repetition number are noted (for example, “1_1” means training session 1, repetition number 1).

During the training period of four weeks, the time taken for completion of each task repetition was noted at every training session. The mean time to task completion for the peg transfer task at each repetition is shown in Figure 1 for both groups. The learning curves in both groups were very similar. At the beginning of every session, the time for task completion tended to be higher than the value achieved at the last repetition of the previous session, with the performance improving again at the subsequent repetitions of each session (Figure 1).

### 3.6. Side Effects Related to 3D Visualization

None of the participants of our study experienced nausea, dizziness, or any other symptom related to 3D visualization.

## 4. Discussion

In this randomized controlled trial, training of BLS on a standard laparoscopic box trainer using standard 2D visualization was found to be at least equally effective compared to 3D in terms of skills improvement. After a training period of four weeks, a significant improvement of skills was observed in both study groups, irrespective of the type of visualization used.

Non-inferiority of 2D compared to 3D could be shown for total score improvement and for score improvement of the single FLS tasks (peg transfer, pattern cutting, and suturing with intracorporeal knot tying task) except for the suturing with extracorporeal knot tying task. In the case of peg transfer score improvement, even superiority could be shown for the 2D compared to the 3D group. This might be explained by the fact that at the baseline test, participants in the 3D group scored better than those in the 2D group at this task and therefore also had higher total scores, whereas the other task scores did not differ between groups. However, during the training period, 2D group participants caught up on this initial deficiency with a greater improvement than the 3D group resulting in no score difference at the post-training test between groups.

When taking together all four FLS tasks, superiority in total score improvement could not be seen, but only a tendency to a greater score improvement in the 2D compared to the 3D group (*p* = 0.055, Table 3). However, this may be due to the artificially increased SDs of the total score improvements caused by the “0-scores” at the suturing with extracorporeal knot tying task of participants with drain avulsion. When this task was excluded from the analysis, superiority in total score improvement could indeed be seen, which seems to better reflect the real situation.

At the post-training test using the assigned visualization modality, there were no differences between groups regarding any of the four evaluated tasks.

These results suggest that for novices without previous laparoscopic experience, at the beginning, 3D may offer a slight advantage over 2D. In the present study, this was observed only for the peg transfer task, which could be more dependent on good depth perception than the other tasks. For the suturing tasks, needle handling as well as suturing and knot tying skills seem to be more pivotal for time for task completion and accuracy than depth perception. However, during the training period, participants seemed to adapt to the 2D perception of the 3D environment, thus catching up with the participants of the 3D group, until reaching comparable skill levels at the end of the training period, as shown by the non-inferiority of the 2D group in test score improvement and comparable post-training test scores.

FLS tasks were chosen because an objective scoring system for these tasks was previously published, the results can be compared to existing literature and the validity of these tasks was thoroughly evaluated [15,16,17,18]. We used two easier tasks (peg transfer and pattern cutting) and the two more complex suturing tasks to evaluate different levels of task difficulty.

For baseline and post training tests, the FLS scoring system was used to assess individual task performance, as previously described [13]. For each task, the time needed for task completion was measured and the accuracy of task performance was evaluated, taking into account different parameters according to the respective task. In the case of both suturing tasks, the FLS scoring system set the assignment of 0 points, if the drain on which the suture had to be made was avulsed. This 0-point scoring is suitable for an exam setting where participants can either pass or fail and a further evaluation is not relevant. However, if performances have to be analyzed in a continuous manner and not only with regard to a passed/failed status, it is important to have a more detailed gradation also in the “failed fraction”. For future studies, it might be useful to consider this issue and possibly develop an additional more “gradual” scoring system in case of drain avulsion, taking into account accuracy and time taken until avulsion occurs, and possibly also continuing the task in some form on a new drain to evaluate the rest of the task.

After the first post-training test using the assigned modality, a second post-training test was carried out using the modality that was not used during training. When considering the challenge of a reduced depth perception of the 2D visualization, we hypothesized that participants of the 2D group may achieve better scores when changing to 3D even if they are unfamiliar with it, and that participants of the 3D group, conversely, may have more difficulties when changing to 2D, and therefore, show a worsening in test scores. However, no significant changes, neither in total scores nor in individual task scores were found when carrying out the post-training test using the “extraneous” modality. In the 3D group, only at the suturing with extracorporeal knot tying task and consequently for the total score, significantly better scores were seen at the 2D compared to the 3D post-training test. However, this seems to be due to the fact that drain avulsion occurred at the first test (the 3D test) of this group with resulting “0-scores” for this task, for those participants who did not repeat drain avulsion at the second test (2D test). When excluding this task from the analyses, no difference in any of the scores was found anymore.

As the second task repetition usually showed a better score than the first one at each training session (Figure 1), a possible worsening in the test scores of the 3D group when changing to 2D might have been counteracted by the fact that the 2D post-training test was performed right after the 3D test in this group and therefore represented the second task repetition on that day. Regarding the 2D group, it seems that the unfamiliarity with 3D was more decisive than its advantage of a better depth perception for the post-training scores, resulting in comparable test scores to those achieved using 2D. However, overall, a change from the own, “familiar” visualization modality to the other one did not cause any worsening in BLS performance, underlined by the fact that the scores achieved at the 2D post-training test were comparable between groups, and the same was true for 3D post-training test scores. Therefore, BLS may be trained using either modality without having any disadvantages when changing to the other one.

In this study, it was decided to work with a fixed camera in order to avoid possible bias arising from differences in camera navigation. However, this could also be considered as a limitation of the study, as the learning curve for camera navigation was not evaluated and possible side effects of 3D visualization, such as dizziness and nausea, might have been missed due to their possible relation to camera motion. Another limitation is that except for one participant, all students were right-handed. If dizziness and nausea are more prevalent in left-handed persons, the evaluation of mainly right-handed participants might have contributed to the fact that no such side effects of 3D visualization were observed in this study. Furthermore, only BLS were evaluated and participants had no significant laparoscopic experience. Therefore, the present results may be generalized to the BLS performance of trainees with no or little experience in laparoscopic surgery. However, such trainees or even more experienced surgeons might take advantage of 3D visualization when performing more advanced tasks or complex surgeries, as shown in gastric bypass procedures or complex hysterectomies for large uteri [6,7]. On the other hand, more experienced surgeons may already have adapted so well to 2D vision that the advantage of better depth perception through 3D vision may not lead to a substantial benefit. In this regard, previous studies suggest that 3D may be more beneficial for less experienced trainees [5,19,20]. However, at least one study in the educational setting evaluating laparoscopic vaginal cuff closure during total laparoscopic hysterectomy by surgeons-in-training could not show any significant difference between 2D and 3D vision in the time needed for completion of this surgical step, and also the surgeons’ subjective rating of the “ease of cuff closure” did not differ between groups [8]. Taken together, further studies evaluating more complex tasks and surgeries, objective outcome measures, and differentiating the results based on the participants’ surgical experience are needed.

## 5. Conclusions

Training using conventional 2D visualization may be considered to be at least equally effective compared to 3D in terms of BLS improvement, while having the advantage of being less expensive and already extensively adopted in hospitals. Furthermore, it seems that BLS may be trained using either visualization modality without having any significant disadvantages when changing to the other modality.

## Figures and Tables

**Table 1:** Baseline demographic data of participants in the two-dimensional (2D) and three-dimensional (3D) groups.

	2D (*n* = 16)	3D (*n* = 15)	*p*-Value
**Year of medical studies** [years], median (IQR) ^1^	4 (4.5–5)	4 (4–5)	0.470
**Sex**, female:male	10:6	4:11	0.073
**Handedness**, right:left	16:0	14:1	0.484
**Assisted in laparoscopic surgeries**, yes:no	6:10	5:10	1.000
**Box trainer experience**, yes:no	0:16	2:13	0.226
**Video game experience**, yes:no	5:11	6:9	0.716
**Played a musical instrument**, yes:no	7:9	5:10	0.716
**Virtual reality trainer experience**, yes:no	0:16	0:15	1.000

^1^ IQR: interquartile range. Mann–Whitney–U test was used for comparisons of ordinal data. Nominal data were compared using the Fisher exact test; *p*-Values < 0.05 were considered statistically significant.

**Table 2:** Comparison of the baseline and post-training test scores for the total scores as well as for the four different Fundamentals of Laparoscopic Surgery tasks (peg transfer, pattern cutting, suturing with extracorporeal knot tying, and suturing with intracorporeal knot tying) within and between groups using the respective assigned visualization modality.

	2D Median (IQR) ^1^	3D Median (IQR) ^1^	*p*-Value (between Groups)
**Total score**			
Baseline test score	13.0 (0.5–34.9)	37.5 (5.8–64.6)	0.033 *
Post-training test score	303.9 (276.2–313.1)	285.3 (250.0–328.0)	>0.999
*p*-Value (within the group)	<0.001 *	0.001 *	1.000
**Peg transfer**			
Baseline test score	0 (0–4.2)	14.1 (0–19.8)	0.019 *
Post-training test score	77.0 (73.4–84.1)	78.1 (73.0–84.0)	0.953
*p*-Value (within the group)	<0.001 *	0.001 *	-
**Pattern cutting**			
Baseline test score	0 (0)	0 (0–15.4)	0.232
Post-training test score	56.0 (53.6–61.1)	57.4 (48.5–65.0)	0.922
*p*-Value (within the group)	<0.001 *	0.001 *	-
**Suturing with EC** ^2^			
Baseline test score	0 (0–10.6)	0 (0–6.7)	>0.999
Post-training test score	82.9 (70.2–93.5)	78.8 (35.0–92.3)	0.520
*p*-Value (within the group)	0.001 *	0.002 *	-
**Suturing with IC** ^3^			
Baseline test score	0 (0–9.0)	5.8 (0–47.5)	0.188
Post-training test score	85.6 (83.0–86.8)	84.8 (79.4–93.8)	0.953
*p*-Value (within the group)	<0.001 *	0.001 *	-

^1^ IQR: interquartile range; ^2^ EC: extracorporeal knot; ^3^ IC: intracorporeal knot. The Wilcoxon-signed-ranks test was used for comparisons within groups and the Mann–Whitney–U test was performed for comparisons between groups. * Statistically significant values (*p* < 0.05).

**Table 3:** Comparison of the score improvements for the total scores and the four different Fundamentals of Laparoscopic Surgery tasks (peg transfer, pattern cutting, suturing with extracorporeal knot tying, and suturing with intracorporeal knot tying) between groups using the respective assigned visualization modality.

	2D mean ± SD ^1^	3D mean ± SD ^1^	*p*-Value	NIM ^2^ [pts] ^3^	95% CI ^4^
**Total score**	272.6 ± 34.6	233.9 ± 65.6	0.055	−23.4	−0.9–78.2 *
**Peg transfer**	74.6 ± 8.3	64.0 ± 16.3	0.034	−6.4	0.9–20.4 *^#^
**Pattern cutting**	54.5 ± 9.5	49.0 ± 11.5	0.153	−4.9	−2.2–13.3 *
**Suturing with EC** ^5^	66.5 ± 30.5	59.1 ± 43.5	0.587	−5.9	−20.1–34.8
**Suturing with IC** ^6^	76.9 ± 15.1	61.8 ± 34.5	0.135	−6.2	−5.1–35.3 *

^1^ SD: standard deviation; **^2^** NIM: non-inferiority margin; ^3^ pts: points; ^4^ CI: confidence interval; ^5^ EC: extracorporeal knot; ^6^ IC: intracorporeal knot. The improvement in test scores was calculated as the difference between post-training test scores and baseline test scores, using the respective assigned visualization modality for each group. The *p*-values and two-sided 95% CIs for the *t*-test for independent samples are given. * Non-inferiority of the 2D compared to the 3D visualization modality could be shown. ^#^ Superiority of the 2D compared to the 3D visualization modality.
